# ‘He who knows it, feels it.’ Stepping stones towards fulfilling a peer-advocate role among people with chronic stigmatising conditions in Liberia

**DOI:** 10.1093/inthealth/ihaf126

**Published:** 2026-07-03

**Authors:** Hannah Berrian, Rosalind McCollum, Wede Seekey, Tiawanlyn G. Godwin-Akpan, Zeela Zaizay, Sally Theobald, Laura Dean

**Affiliations:** a University of Liberia, Pacific Institute for Research and Evaluation, Monrovia, Liberia; b Liverpool School of Tropical Medicine, Liverpool, UK; c American Leprosy Missions, Accra, Ghana; d Actions Transforming Lives, Monrovia, Liberia

**Keywords:** mental health, neglected tropical diseases, peer advocate, peer support, stigma, stigmatising conditions

## Abstract

**Background:**

People affected by skin neglected tropical diseases (NTDs) are often stigmatised, experiencing discrimination in consequence of their condition. Peer advocates can help progress a person-centred agenda for people affected by NTDs, however, evidence gaps in the steps to becoming an advocate persist.

**Methods:**

We wanted to understand the steps towards a peer advocacy role for skin NTDs. We used the participatory Stepping Stones method to identify steps on the journey to becoming an advocate with 48 participants, followed by three in-depth life histories with peer advocates for other stigmatising chronic conditions (e.g. disability, mental health, Ebola survivors).

**Results:**

We found study participants frequently experience stigma and discrimination in consequence of their condition. The peer advocate role includes awareness raising and advocacy and providing mutual peer support. Among peer advocates, personal acceptance of their condition, vocational training and family support are important steps along the pathway to becoming a peer advocate. Outcomes of the role include personal determination and increased confidence for peer advocates, provision of help and support for others with similar conditions and broader change in understanding, attitudes and policy within the community and country.

**Conclusions:**

We find that when appropriately enabled, peer advocates can themselves be empowered and play a key role in person-centred care.

## Introduction

People affected by skin neglected tropical diseases (NTDs) (such as yaws, Buruli ulcer, leprosy, lymphatic filariasis and onchocerciasis) are often stigmatised and experience discrimination and isolation as a consequence of their condition.^1–3^ The World Health Organization (WHO) NTD Roadmap 2030 highlights the need for integrated approaches that address the long-term needs of people affected, including mental health and social support.^4^ NTD programs are recommended to ‘incorporate interventions aimed at reducing stigmatisation and breaking down barriers to timely access to care and treatment for individuals, families, communities and marginalized groups’.^4^ Thus peer support has the potential to play a key role in the ongoing care of people affected by skin NTDs through its strong emphasis on empowerment, mental health and social connectedness.^5,6^ Within the strategic framework for integrated control and management of skin-related NTDs, the WHO emphasises actions with potential to be included within peer support interventions, such as self-care to maintain health, prevent disease, maintain health and cope with illness and disability; mental health interventions; stigma reduction; inclusion and human rights.^7^ However, little is known regarding what helps and hinders persons affected to undertake peer support roles and their influence on the empowerment process.

Stigma and discrimination are shared experiences for people affected by NTDs, Ebola virus disease (EVD), physical disability and/or mental health conditions (MHCs).^8–10,26–28^ For example, persons affected by NTDs and/or MHCs may be deprived of job, education or marriage opportunities due to stigmatisation and discrimination.^7,10^ Despite growing evidence regarding the use of peer support among people affected by skin NTDs,^11^ peer support approaches have been more widely used among people with MHCs.^12,13^ Peer support is defined as ‘a system of giving and receiving help that is founded on key principles of respect, shared responsibility and mutual agreement of what is helpful’.^5^ The shared experience of emotional pain often experienced by patients with a common health condition creates affiliation and a sense of connection that enables peers to ‘be with each other without the constraints of traditional (expert/patient) relationships’.^5^ Similarly, Puschner et al.^6^ highlight the patient-centred nature of peer support, emphasising the importance of empowerment and social inclusion rather than just clinical outcomes. The peer advocate role supports recovery of the peer advocate and others through practical and emotional support, empowerment and widening social networks.^6^ This understanding presents an opportunity for the NTD community to learn from other sectors. By centring lived experiences of peer supporters/advocates from other sectors, we can collectively design peerled interventions for use in NTDs that can progress a person-centred agenda.

Skin NTDs (Buruli ulcer, yaws, onchocerciasis, leprosy and lymphatic filariasis [resulting hydrocele and lymphoedema]) are high among the general population in Liberia.^14,15^ In 2016 Liberia became one of the first countries in the world to introduce integrated case management for NTDs into its health system. Reducing the burden of severe stigmatising skin diseases in Liberia (REDRESS) is a Liberian Ministry of Health (MOH)-led research program that aims to strengthen integrated case management and improve care for people affected by skin NTDs, including the establishment of peer support groups. When this study commenced, there were limited established peer support groups among people affected by skin NTDs in Liberia. There is also limited evidence regarding the steps in the journey towards becoming a peer advocate, particularly for NTD peer advocates. In response, we engaged with peer advocates for other chronic health conditions (with existing established networks) to learn from their experiences and inform intervention design using participatory methods to explore their journey to advocacy and prioritising them as co-producers of knowledge.

## Methods

### Study design

**Figure 1:**
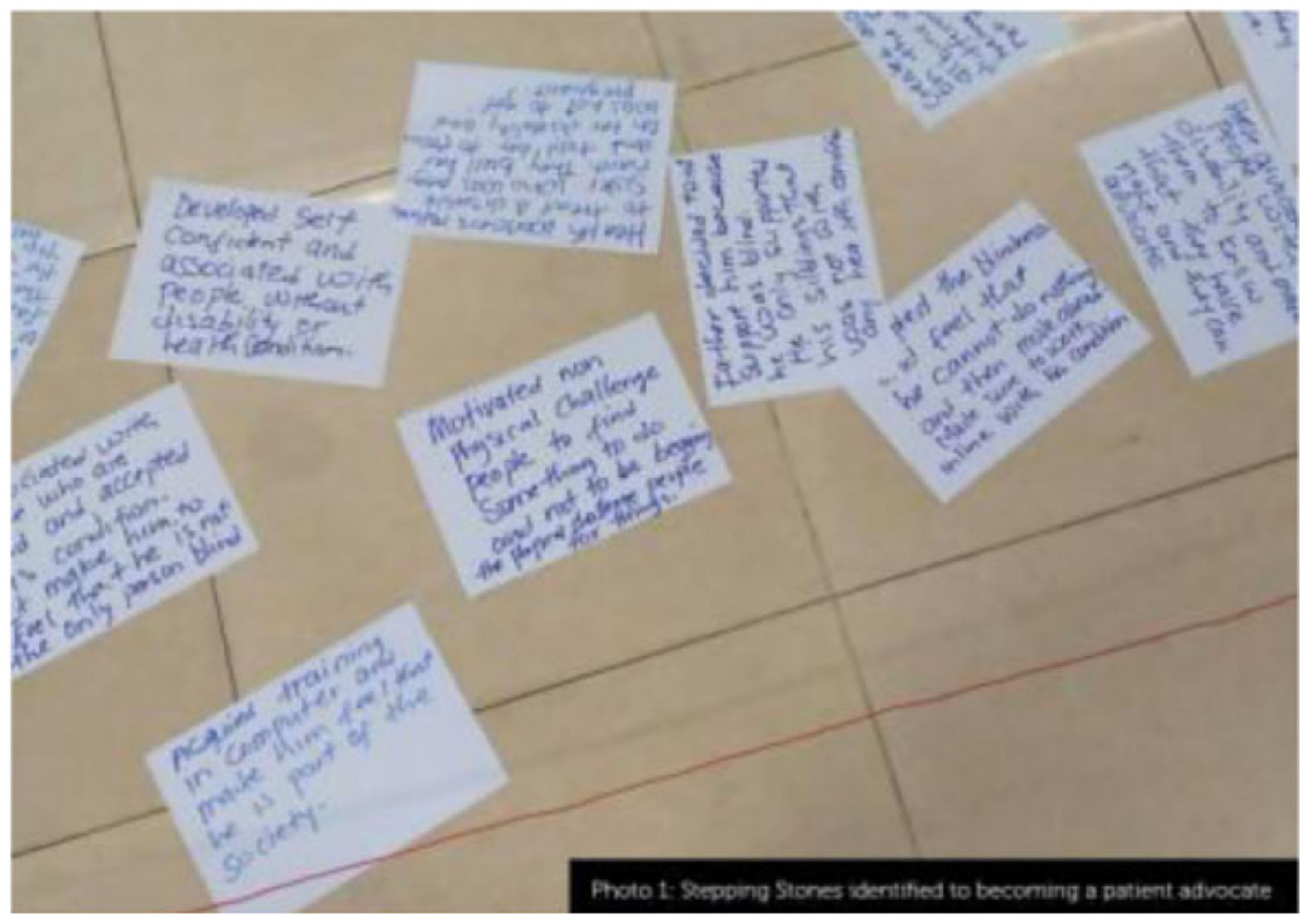
Stepping stones identified to becoming a patient advocate. Photographer Hannah Berrian.

**Figure 2:**
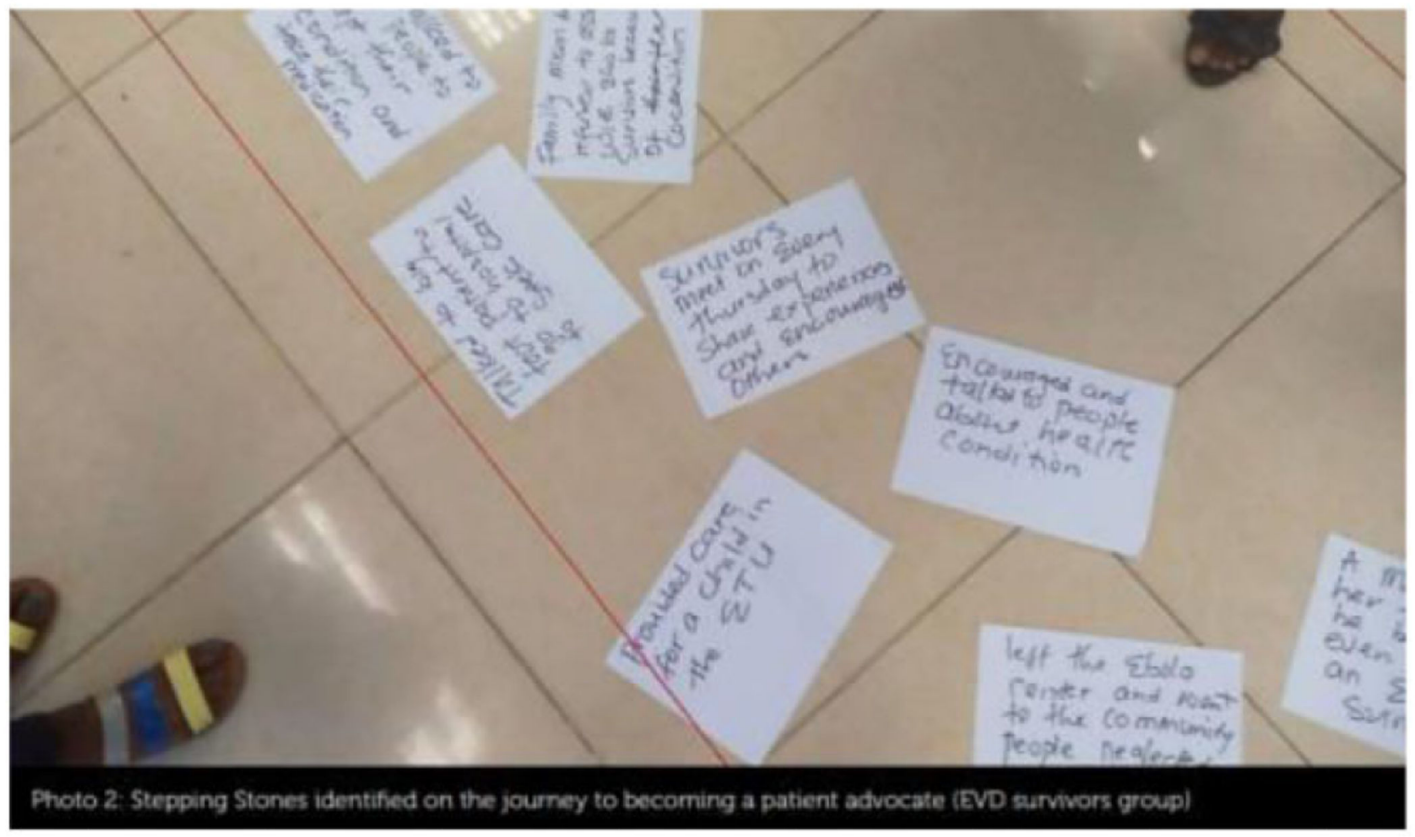
Stepping stones identified on the journey to becomig a patient advocate (EVD survivor group). Photographer Hannah Berrian.

This study was situated within the naturalistic paradigm seeking to give emphasis to and understand the experiences and views of peer advocates living with a stigmatising condition.^16^ We used qualitative methods, including several participatory methods, to gather in-depth information on the pathway to fulfilling a peer advocate role. Stepping Stones and life histories were used to explore with participants enabling steps along their journey to becoming a peer advocate. Stepping Stones involves peer advocates and researchers jointly determining what steps are needed to cross a fictional river to achieve an outcome^17^ (see Figures 1 and 2).

During life history interviewing, people are asked to provide a personal account of their life in their own words and according to their personal timelines, marking key life events including education, illness or death within the family, marriages, births etc.^18^

### Study context

Primary data were collected from participants in Monrovia during March 2021 as part of the broader REDRESS program. The peer support networks are headquartered in Monrovia, which was most convenient for selected participants.

### Participants and sampling strategy

Six Stepping Stone workshops were facilitated involving 48 peer advocates living with other stigmatising conditions. Participants were selected through a purposive selection technique to ensure maximum variation in age, gender, health condition and previous involvement with peer support groups and/or advocacy activities. To identify potential participants, we engaged pre-existing peer advocate groups (including Cultivation for Users Hope [CFUH], Ebola Survivors Network and National Union of the Disabled [NUOD]), inviting people who have (or who have had) disabilities, MHCs or EVD. Participants were included on the basis of being part of a peer advocate group and having one of the chronic stigmatising conditions. Participants < 18 y of age or unable to provide informed consent were excluded. Written informed consent was obtained from each participant.

During the Stepping Stones workshops, we identified three participants who are particularly active peer advocates with a leadership role within existing networks, inviting them to take part in the life history interview. Table 1 summarises the study participants.

With consent, qualitative data were audio recorded and transcribed verbatim. Quality checks were carried out for several transcripts to ensure quality. An inductive thematic framework approach to analysis was used.^19^ The researchers reviewed and familiarised themselves with the data transcripts, following which data were analysed using Padlet software (https://padlet.com/) to allow for online collaboration. Ideas were identified and codes were developed. These were jointly reviewed by the researchers and organised based on arising themes using a framework approach, with illustrative quotes identified.^20^

## Results

**Figure 3:**
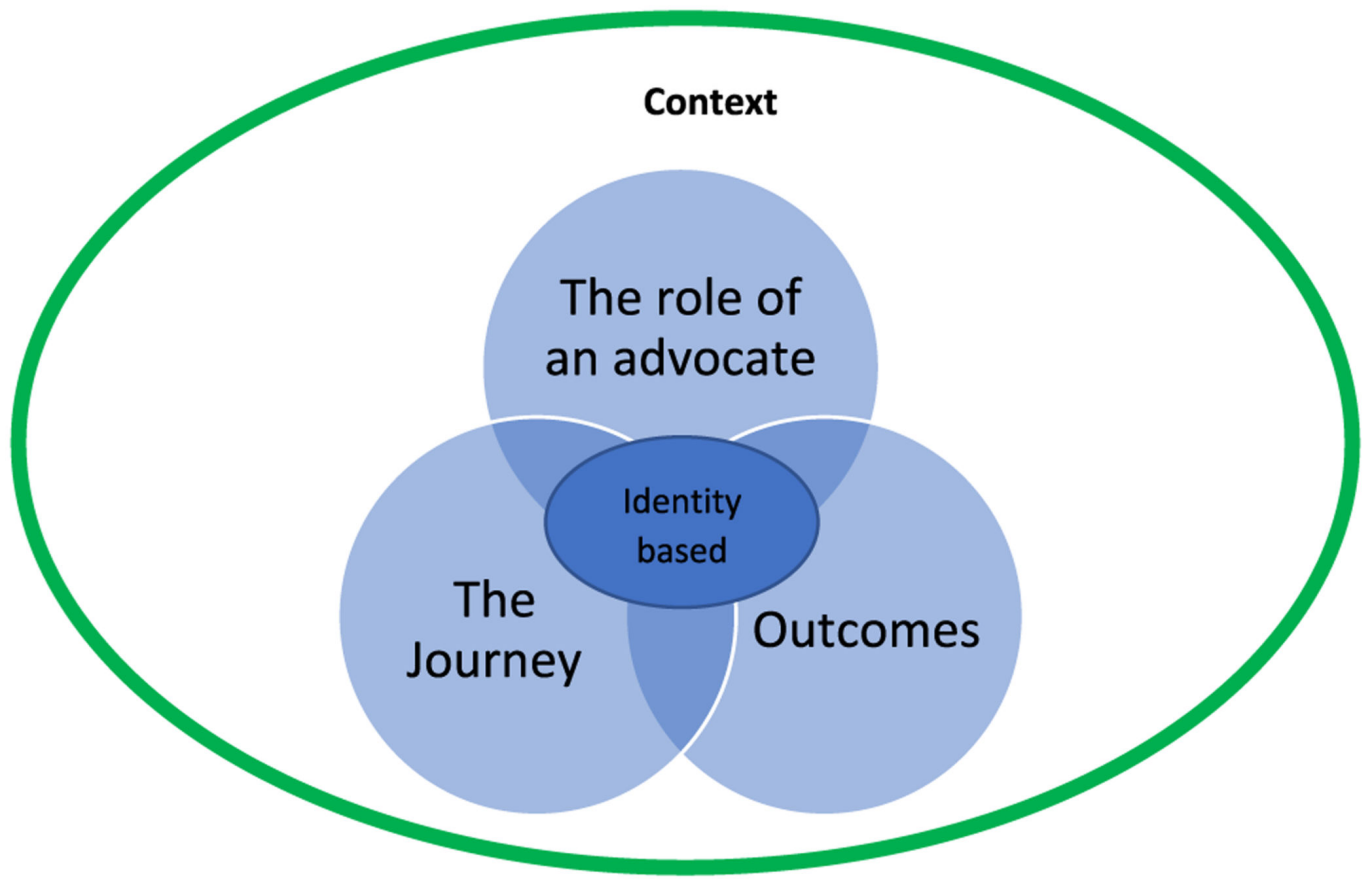
Identified themes.

Three main themes emerged from the findings—the role of an advocate, the journey to becoming an advocate and the outcomes—all positioned within the local context with strong reliance on informal providers such as herbalists and faith healers prior to formal health seeking (Figure 3), as well as their experience of stigma and discrimination, including the ending of relationships, neglect by parents, loss of home, rejection by their community, challenges being allowed to attend school and difficulty finding a job.

### The role of an advocate

All participants described key aspects of their role as a peer advocate, which required building on pre-existing community relationships including awareness raising and advocacy and taking part in peer support groups.

### Advocacy and awareness

Most participants described involvement with awareness-raising activities about their condition, including the causes of the condition and the importance of regular treatment for management.

So, in my environment when I saw other people living with this condition [MHC]…what I learned from my health workers I decided to explain it to the other family members…I also carried on awareness to encourage other people that anybody can live with this condition and live a normal life.

— Women with MHC group

Both men’s and women’s Stepping Stones groups highlighted the importance of advocacy in both community and with government to promote health and welfare needs of people affected, including support for mental health issues, the need for a sign language interpreter at the hospital, ramps for wheelchair users at the hospital and fair justice for people with disability in court.

If you don’t tell anybody about your problem they will not know, so we have been doing that awareness and telling people about the United Nation Convention on the Rights of People with disability (UNCRPD).

— NUOD women’s group

Challenging experiences/denial of rights motivated some participants to challenge legal or administrative norms through their advocacy roles, e.g. seeking to become a district legislator to have input in decision-making and ensure allocation of funds for EVD survivors.

…to venture into politics could help me advocate for the survivors.

— Male EVD life history participant 03

### Peer support

Peer support was discussed by the majority of participants, particularly if they experienced isolation from their family. Women, in particular, described how enjoying time to joke and laugh together was central to bringing normality, hope and strength to their support group.

All survivors will come here and we agreed upon it [meeting together] for every Thursday. As we get there as you meet your friends we laugh, play, joke and then everything became or started getting normal again.

— EVD survivor women’s group

Participants with MHCs in both groups and life history described establishing peer support cell groups within their respective counties. Sharing stories and testimonies together during peer support groups based on their common experience brought encouragement and helped address stigma, since ‘he who feels it, knows it’ (EVD survivor men’s group).

The mental illness group helps us a lot. Yes, they help a lot. They encourage us.

— Women with MHC group

### Journey to fulfilling the peer support role

**Figure 4:**
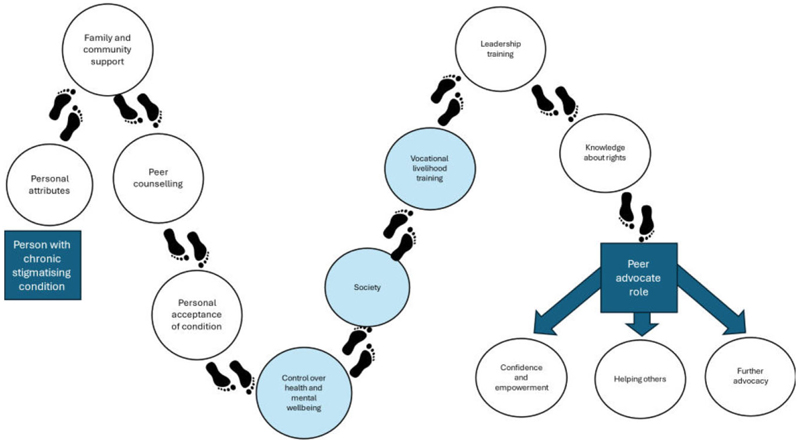
Stepping Stones to fulfilling a peer advocate role. Steps in blue correspond with priority areas in the WHO strategic framework for integrated control and management of skin-related NTDs.

Figure 4 presents an illustrative synthesis of the stepping stones identified along the path to becoming peer advocates. Personal attributes such as determination and education already existed and contributed to an individual’s journey. While there was some variation, the most commonly described steps along the journey to becoming an advocate included family support, peer counselling, personal acceptance of their condition and taking part in vocational and leadership training with realisation of rights.

#### Personal attributes—determination and education

Women’s groups described a need to be persistent, determined and to have self-motivation, particularly when experiencing discrimination. The education history of participants provided an important basis for the journey towards becoming a peer advocate, with persons with higher levels of education more likely to describe becoming an advocate.

#### Family and community support

The importance of support from family and friends was widely described. Regardless of the underlying condition, participants emphasised the importance of encouragement, caring and being able to talk with their family and friends in creating stability for their recovery and contributing towards their own personal acceptance.

During the course of my illness, some 30 years from 1976 to 2006, my father was my carer and after my father passed in 1992 my elder brother took over. When my eldest brother passed, my wife became my caregiver. So these are people who took care of me and who made sure that my life was not taken advantage of.

— Life history MHC participant 01

#### Peer counselling

The need for counselling was described by almost every Stepping Stones group, including formal door-to-door counselling and informal talking and encouraging others. Participants felt drawing upon their own experiences was useful when counselling others.

You tell the person your experience first so the person can know that you were in that situation. From there you talk to him, through your explanation and other things that you went through, that person may understand.

— Men with MHC group

#### Personal acceptance

Two of the men’s Stepping Stones groups spoke about the importance of personal acceptance of their condition as being the first important step in their personal journey. Both the men and women’s MHC Stepping Stones groups and the women’s EVD group highlighted the importance of taking medications regularly as contributing towards creating a sense of control over their own well-being.

If you accept your particular condition at that particular stage, then you can be able to bring anybody on board. If you don’t accept your condition, then it will be difficult to recruit you. So, first you have to accept your condition.

— EVD survivor men’s group

#### Social capital

Two life history participants described how their motivation to be peer advocates was linked with their personal experiences of having been stigmatised previously, e.g. having been excluded from attending school, being unable to work and isolation from family and the wider community.

Well because I got mental illness and they didn’t trust me going back amongst the other good kids and so I was asked to stay away from school.

— Male MHC life history participant 01

#### Training and realisation of rights

In supporting individuals to transition to the role of peer advocates, provision of training or skill-building activities is essential. Male peer advocates in particular emphasised the importance of training for people affected, including condition-specific advocacy training, peer specialist training, leadership and proposal writing training, promoting hope and self-respect.

Through the [training] intervention…That really gave me the encouragement, because I felt to myself really I wasn’t going be somebody in society, but through the two-week programs that we had at that time we were encouraged.

— CFUH men

Alongside this, participants highlighted the need for vocational training, particularly for those with MHCs, so they can provide for themselves since stigma may create a barrier to their employment. Vocational training described included tailoring, soap-making, arts and crafts, tie dying, agricultural activities, computers, beauty care and garage mechanic. There were some gendered differences in the types of vocational training between men and women, linked with gendered roles and masculinities, e.g. women were more likely to describe beauty care training, with men more likely to describe garage mechanic training. Education and training were viewed as critical to the realisation of rights.

I knew because of the illness I suffered I was not going to work for anybody because of the stigma associated with the kind of illness that I suffered. So we were trying to capacitate ourselves so that wherever we go we can be independent.

— Male MHC life history participant 01

### Outcomes of advocacy role

#### Empowerment through increasing confidence

Women’s EVD survivors and the NUOD men’s group both spoke about gaining confidence through their advocacy role, including associating with people without disability or a health condition, enabling them to overcome stigmatisation. Holding responsibilities through their role created a sense of empowerment.

…that they have empowered some of us and today they can give some of us responsibilities. Today I have a responsibility, to talk to people, to convince people, to educate people, to bring people back on board.

— Men with MHC group

#### Helping others affected

Both men and women spoke about their responsibility to help others. There was a difference in how this was described between men and women. Women describe an informal role, encouraging others to seek care, take treatment and avoid isolation.

…by you going to them, talking to them, encouraging them, even sit with them that will give them some light to bring back.

— EVD survivor women’s group

In contrast, men described taking part in more formalised ‘helping’ activities, such as counselling survivors at the Ebola Treatment Unit (ETU), providing support for those living on the street. It may be that for some of these roles there is financial incentive provided, while other activities are likely to be voluntary (although this was not explicitly probed).

#### Bringing wider advocacy change within the community and country

All life history participants described having been involved with advocacy work. Participants with MHCs described advocating for budget allocation and the Mental Health Act (one participant only). Advocacy methods described include radio and international conference attendance to raise the profile for mental health globally.

## Discussion

Our study found that people living with chronic health conditions in Liberia frequently experience stigma and discrimination. We found there are a range of peer advocate roles, including awareness raising and advocacy and providing mutual peer support. Critical steps on the journey to becoming a peer advocate include family support, peer counselling, personal acceptance of their condition and taking part in vocational and leadership training to support realisation of rights (see Figure 4). In response, RE-DRESS worked together with persons affected by skin NTDs to co-develop a peer support intervention that led to a series of interventions. This is described elsewhere.^21^

### Peer support roles

The roles identified in our study have clear similarities with those in the mental health literature, such as helping peers; providing social support, engagement, counselling and emotional support; inspiring hope for others affected, as well as education/awareness-raising roles.^6,13^ Personal support interventions (including peer groups) have been identified as one of five priority interventions for the reduction of NTD-related stigma.^10^

### The journey towards a peer support role

The stepping stones identified from our study have many similarities with the WHO guidance for empowering persons affected by skin NTDs at the community level.^7^ Stepping stones highlighted in pale blue in Figure 4 are the four priority areas (health, society, mental well-being and livelihood) defined in the WHO strategic plan, confirming the importance of these areas for peer advocates with a range of conditions.

Personal attributes play a role in influencing a person’s progression towards fulfilling a peer advocate role. In our study, some participants described drawing upon self-motivation and determination, perhaps in response to experience of stigma, in keeping with findings by De Beer et al.^13^ Education varied considerably among study participants. Prior literature revealed mixed findings surrounding prior levels of education for peer advocates, with some studies stressing the need for a certain prior level of education, while other studies emphasise that skills needed may differ from those gained through education.^13^ Due to the chronic nature of skin NTDs and the limitations some affected persons encounter to complete their education, it is critical that actions to empower affected people benefit all and do not lead to a widening gap between a minority of people who have already benefited from a more comprehensive education and the majority of people affected who may have faced barriers to even basic education. Community engagement and participatory methods are useful ways to ensure meaningful engagement of affected persons as ‘experts by experience’ in research.^22^

Caring support from family, a recurring enabler throughout the patient journey, is also strongly emphasised in the NTD literature.^7,10^ Koschorke et al.^10^ highlight the relationship between family and stigma for people affected by NTDs, with family members at times stigmatising or themselves experiencing stigma and discrimination in consequence of their relationship with people affected by NTDs.

Our findings reveal the importance of strong prior relationships between peer advocates and community, who may subsequently become their clients/peers. A previous study found the need for strong non-judgemental relationships with the ability to maintain boundaries as important for peer advocates.^23^ This may be particularly important within peer support groups where there are people affected by a range of skin NTDs, given prior evidence of ‘interdisease stigma’ between people affected by leprosy and Buruli ulcer receiving integrated care in Liberia.^23^

We found strong emphasis on personal acceptance and coming to terms with their condition, similar to findings about the transition from service user to peer support worker among youth with lived experience of mental illness.^13^ Within the NTD literature, Dean et al.^11^ describe this transition to that of a quest narrative, where illness becomes an integral part of individual identity as well as a motivator for social action and/or change. Counselling by fellow peers, sharing experiences and gaining knowledge and understanding about the condition and gaining control over their physical and mental well-being through consistent access to medication were described as steps on the path towards personal acceptance or a manifesto narrative. The concept of experiencing control relating to experiences was emphasised by De Beer et al.,^13^ with the need to be aware of ongoing experiences of peer support workers, who may re-experience feelings of powerlessness and lack of control if treated unequally with non-peer staff.

The provision of training, both vocational and for a peer advocate role, was widely described as building confidence and empowerment. Bangert et al.^9^ emphasise the economic effect of NTDs for people affected (and their families), due to lost productivity related to disability, disfigurement and debilitating effects (including mental health impact) and stigma-related exclusion from work. We support the recommendation of Koschorke et al.^10^ that policy change relating to social and economic inclusion with a rights-based approach is essential. For peer support roles, training relating to interactions as a peer support worker, including conflict management, engaging and empowering others and having adequate knowledge about the condition, is required.^13^

#### Other potential enablers in the peer support role identified within the literature

The literature reveals two additional themes that enable the peer advocate role that did not emerge strongly from our study but may provide valuable reflections for policymakers developing peer support programs, including:

- The relationship between peer advocates and non-peer staff (formal health workers), e.g. supervision and mentoring, clear role requirements, addressing concerns/negative attitudes between peer advocates and health workers, providing an opportunity to share power,^13^ as well as ensuring health workers have adequate time and guidance regarding peer support.^24,25^
- Organisational readiness, with training for non-peer staff with recognition and appreciation for those with lived experience across the organisation.^13^ Within our study participants described having faced barriers to bringing about meaningful policy change despite extensive advocacy efforts, with limited budget having been allocated towards mental health, despite extensive advocacy work, which may demonstrate a lack of organisational readiness to the peer advocate role.

In light of our findings and the literature, we propose policymakers and planners take into consideration five recommendations when developing peer support groups (see Box 1).

Additionally, we found extremely limited evidence that explored the enablers towards the peer advocacy role among persons affected by NTDs. Future research is recommended to address this gap and explore how best to empower persons affected by skin NTDs to take on peer support roles.

### Outcomes of peer support

Participation in peer support was felt by study participants to have contributed towards three main benefits:

- Personal empowerment: Participants described increased confidence and emotional support, in keeping with findings by Trasolini et al.,^25^ which found that peer supporters gained emotional support through their role and increased decision-making confidence.
- Encouragement for persons affected: Participants described providing emotional support and giving hope to others with similar conditions, as well as mobilising practical support. In keeping with chronic kidney disease peer supporters.^24^
- Community awareness: Broader benefits for the wider community, and potentially the country in general, through awareness raising and advocacy. There has been limited discussion of this within the literature to date.

### Trustworthiness and Limitations

The study was carried out among people with other chronic conditions rather than people affected by skin NTDs, due to limited known peer support approaches for people affected by skin NTDs within Liberia at the time the study was carried out. Despite this limitation, our study found remarkable similarities with areas for empowerment identified in the WHO Strategic Framework for integrated control and management of skin-related NTDs, indicating findings are applicable across both groups of persons affected. The participatory nature of the methods used provided an opportunity for active involvement and participation of persons with varied literacy levels. Life histories were carried out with three participants who were most vocal in expressing their experiences during the Stepping Stones discussion. As a result, three well-educated men were selected. While these life histories provided a wealth of valuable information, their experiences are unlikely to be representative of the majority of peer advocates, including women and those with less educational opportunity. In order to avoid overgeneralising findings from an elite group of life history participants, the results made it clear which findings were described by Stepping Stones participants and which were described by life history participants.

## Conclusions

We found that key enablers for peer advocates to fulfil their roles included personal acceptance of their condition, vocational training and support from family. Peer advocates can play a valuable role in supporting person-centred care.

## Figures and Tables

**Table 1:** Stepping Stones and life history participants

Method	Participant description	Participants, n	Gender		Age range (years)	Education
			Male	Female		
Stepping Stones	People with disability -National Union of Disabled	16	8	8	25–50	Mixed literacy levels
	Mental health service user group (cultivation for users hope)	16	8	8	25–50	Mixed literacy levels
	Ebola survivors’ group	16	8	8	25–50	Mixed literacy levels
Life history	Person affected by mental health condition	1	1		40–50	Literate
	Person affected by mental health condition	1	1		40–50	Literate
	Ebola survivor	1	1		30–50	Literate

## Data Availability

Data are presented within the manuscript. Any additional data required can be made available upon request.

## Box 1 Recommendations for developing peer support groups for persons affected by skin NTDs.

1. Create appreciation of lived experiences of people affected by skin NTDs within the MoH through awareness and training across health system levels about peer advocates.
2. Provide adequate training and mentoring for peer advocates to enable them to confidently take on their role.
3. Ensure reliable and consistent supply of medications to enable persons affected to have greater control over their well-being.
4. Educate family members how best to support their loved ones to live with their condition.
5. Provide linkages between peer support groups and external organisations for vocational training to enable empowerment and promote economic independence.
